# Diagnosis of coronary artery spasm by ergonovine provocation test of radial artery

**DOI:** 10.1038/s41598-021-83356-0

**Published:** 2021-02-12

**Authors:** Ye-fei Li, Yu Zhang, Liang Chen, Kou-long Zheng, Hui-he Lu, Zhen-qiang Sheng

**Affiliations:** The Department of Cardiology, the Second Affiliated Hospital of Nantong University, No. 6, Hai’er Xiang North Road, Chongchuan District, Nantong, 226001 China

**Keywords:** Cardiology, Diseases

## Abstract

We investigated the sensitivity, specificity and safety of ergonovine provocation test of radial artery in the diagnosis of coronary artery spasm (CAS). The patients who came to our hospital for chest pain from January to June 2020 as well as had coronary stenosis < 50% and no radial artery stenosis, were enrolled in this study. These patients were divided into CAS group and control group after intracoronary ergonovine provocation test. All patients underwent ergonovine provocation test of radial artery, the inner diameter (D_0_ and D_1_) and the peak systolic velocities (PSV_0_ and PSV_1_) of the radial artery were measured by ultrasound before and after ergonovine provocation. The predictive value of ergonovine provocation test of radial artery for the diagnosis of CAS was analyzed using receiver operator characteristic (ROC) curve. There were 19 patients in the CAS group and 28 patients in the control group. Low density lipoprotein cholesterol and smoking rate were significantly higher in the CAS group than in the control group (all *P* < 0.05), but there were no significant differences in other items (*P* > 0.05) between the two groups. In the ergonovine provocation test of radial artery, degree of radial artery stenosis was significantly higher in the CAS group [41.50% (35.60%, 50.00%)] than in the control group [11.25% (5.15%, 23.00%)] (*P* = 0.000), but there were no siginificant differences in D_0_, PSV_0_ and PSV_1_ between the two groups (*P* > 0.05). The area under ROC curve of ergonovine (120 µg) provocation test of radial artery for the diagnosis of CAS was 0.912 with 95%CI: 0.792–0.975, *P* = 0.001, cut-off of 31%, specificity of 92.86% and sensitivity of 84.21%. The ergonovine (120 µg) provocation test of radial artery did not cause any adverse reactions. We concluded that the ergonovine provocation test of radial artery has high sensitivity, specificity and safety in the diagnosis of CAS.

## Introduction

Coronary artery spasm (CAS) refers to the transient spasm of the main coronary artery or its main branches, which leads to occlusion of the coronary artery and myocardial ischemia. For a long time, people's idea was that myocardial ischemia was associated with coronary atherosclerosis. Coronary angiography is the gold standard for evaluating the severity of coronary heart disease. However, 20-year clinical data challenge this idea, because the coronary angiography indicates normal or almost normal coronary artery in 50% of patients with stable angina and 10–15% of patients with acute coronary syndrome^1^. Nowadays, ischemia with no obstructed coronary arteries (INOCA) has become a research hotspot, and a lot of evidence shows that CAS is one of the main causes of INOCA^2^. CAS is one of the common pathological causes for many ischemic heart diseases, and it can lead to angina pectoris, acute coronary syndrome and even sudden cardiac death^3–7^. Therefore, early and accurate diagnosis of CAS is clinically important.

It is difficult to capture the direct evidence of CAS attack clinically because CAS has the transient characteristic. The sensitivities of non-invasive tests such as 24-h dynamic electrocardiogram, exercise test, hyperventilation test, cold stimulation test and heart imaging are low for diagnosis of CAS. The sensitivity and specificity of intracoronary drug provocation test, an invasive method, are all more than 90%, and the intracoronary drug provocation test has become a gold standard for diagnosis of CAS^8^. The drugs used in the provocation test of coronary artery include ergonovine and acetylcholine. The intracoronary drug provocation test, an invasive method, inevitably cause coronary intervention-related complications. If CAS does not relieve consistently during the intracoronary drug provocation test, intracoronary injection of nitroglycerin is required. Preoperative temporary pacemaker implantation is necessary for dealing with transient atrioventricular block which may occur in intracoronary acetylcholine provocation test. The intracoronary drug provocation test may lead to severe complications such as ventricular tachycardia, ventricular fibrillation, bradycardia, cardiac shock, pericardial tamponade, acute myocardial infarction and death^9^.

The intracoronary drug provocation test requires high conditions and is expensive. Therefore, it is necessary to find a method which has safty as well as similar sensitivity and specificity to the intracoronary drug provocation test, and is easy to carry out in clinical practice. Under pathological conditions, blood vessel dysfunction not only is confined to a particular vessel, but also occurs in systemic vessels^10^. This makes it possible to evaluate coronary artery function by peripheral arteries. Compared with the intracoronary drug provocation test, provocation test of peripheral artery is more safe and simple, so it is a feasible method to use peripheral artery as a window to diagnose CAS.

Both radial artery and coronary artery are medium-sized arteries which are prone to spasm^11^. The high frequency probe of color Doppler ultrasound can clearly show the course, inner diameter and blood flow velocity of the radial artery due to its superficial position. In this study, we performed ergonovine provocation test of radial artery to investigate the influences of ergonovine on the inner diameter and blood flow velocity of the radial artery by ultrasound, and to explore the specificity, sensitivity and safety of ergonovine provocation test of radial artery for the diagnosis of CAS, providing a reference for clinical practice.

### Subjects and methods

All study methods were approved by the Ethics Committee of the Second Affiliated Hospital of Nantong University (2019KW005) , and were performed in accordance with relevant guidelines and regulations. All the subjects enrolled into the study gave written informed consent to participate.

### Subjects and grouping

The patients who were admitted in our hospital due to repeated chest pain from January to June 2020, were collected. Inclusion criteria were (1) coronary stenosis < 50% showed by coronary angiography; and (2) no radial artery stenosis confirmed by ultrasonography. Exclusion criteria included (1) age > 70 years; (2) coagulation and/or hematopoietic diseases; (3) surgical history within 8 weeks; (4) severe cardiac insufficiency with left ventricular ejection fraction (LVEF) < 45%; (5) history of myocardial infarction within 6 weeks; (6) the patients with definite hypertrophic obstructive cardiomyopathy or valvular disease; and (7) the patients with a history of syncope or Aase's syndrome caused by bradyarrhythmias. A total of 47 patients who were consistent with the inclusion and exclusion criteria, were enrolled in this study.

The 47 patients all underwent intracoronary ergonovine provocation test. According to the diagnostic criteria of CAS described in the guidelines for the diagnosis and treatment of coronary spasmodic angina pectoris made by Japanese Circulation Society (JCS)^8^, the intracoronary ergonovine provocation test was positive in 19 patients and negative in 28 patients. The 19 patients were served as CAS group, and the 28 patients as control group. All the 47 patients underwent ergonovine provocation test of radial artery.

### General data

Within 24 h after admission, all patients received related examinations including blood pressure, routine blood test, fasting blood glucose, blood lipids, renal function, troponin I and echocardiography.

### Intracoronary ergonovine provocation test

The intracoronary ergonovine provocation test was performed according to the 2013 JCS guidelines for the diagnosis and treatment of coronary spasmodic angina pectoris^8^ following coronary angiography. These patients must not take calcium antagonists and long-acting nitrate drugs within 48 h, and short-acting nitrate drugs within 6 h. Before the intracoronary ergonovine provocation test, intravenous or intracoronary injections of nitroglycerin and other vasoactive drugs must be avoided. First, 60 µg and 40 µg of ergonovine (Chengdu Beite Pharmaceutical Co., Ltd; Chengdu, China) were respectively diluted in 5 ml of physiological saline, and then respectively injected into the left and right coronary artery within 3 min. One minute later, coronary angiography was performed. The interval of ergonovine injection between left and right coronary artery was 15 min. Positive criteria for the intracoronary ergonovine provocation test: (1) localized or diffuse spasm of coronary artery with a stenosis > 90% after injection of ergonovine; (2) chest pain attacks with or without electrocardiogram-ischemic changes during CAS followed by spontaneous remission within several minutes or disappearance after injection of nitroglycerin into the coronary artery.

### Ergonovine provocation test of radial artery measured by ultrasound and performed through the brachial access

**Figure 1:**
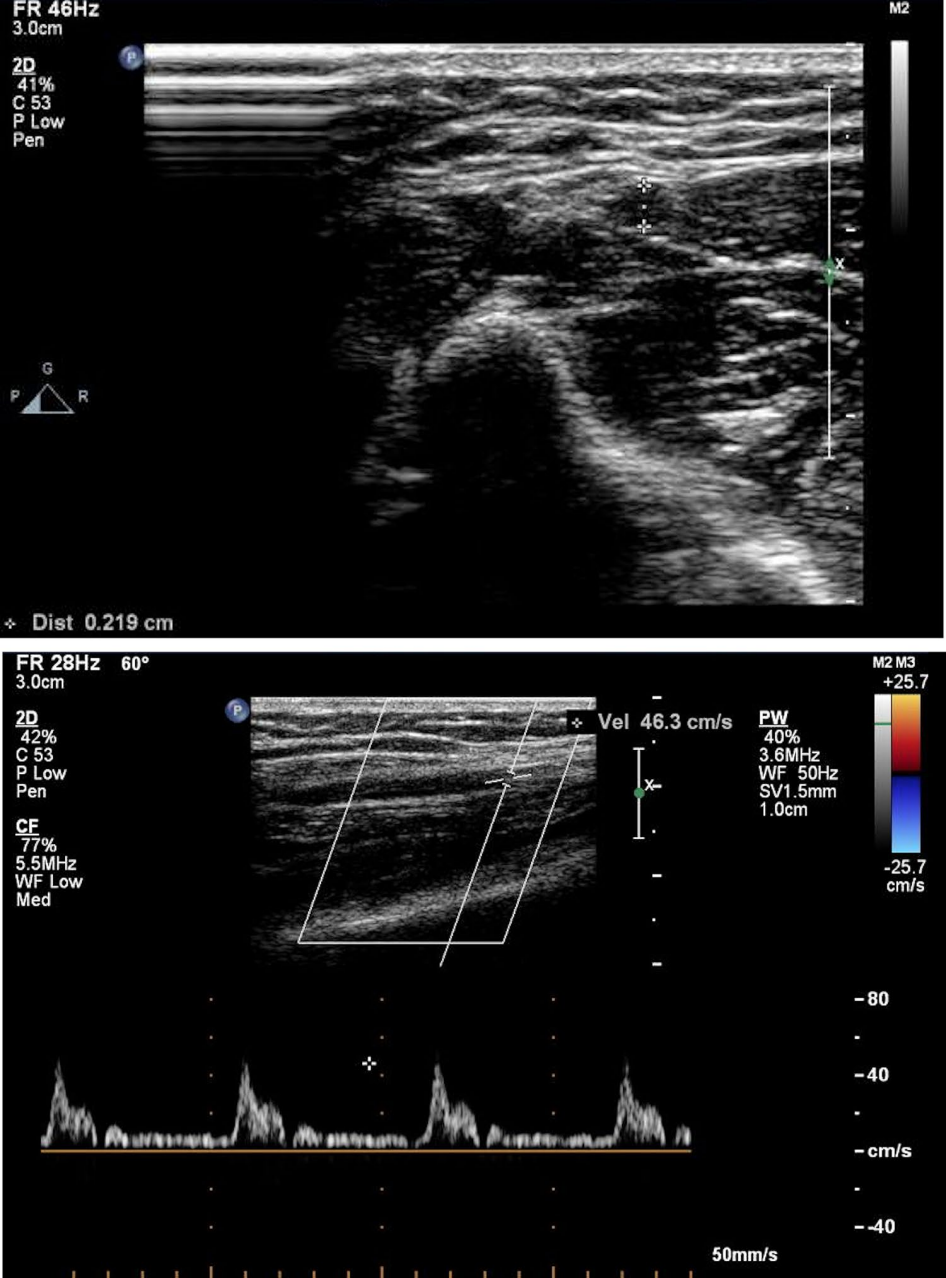
Ergonovine provocation test of radial artery measured by ultrasound. (**A**) Short axis view indicating the inner diameter of the radial artery. (**B**) Spectral Doppler indicating the peak systolic velocity of the radial artery.

Ergonovine provocation test of radial artery was performed more than 15 min after the intracoronary ergonovine provocation test. We used PHILIPS IE33 color Doppler with a probe of L11-3 and a frequency of 5.5 MHz to measure the inner diameter (D_0_ and D_1_) and the peak systolic velocities (PSV_0_ and PSV_1_) of the radial artery before and after ergonovine provocation. The probe was first placed in the right elbow joint to find the radial artery bifurcation, and then was moved along the course of the radial artery to the 5 cm away from the transverse wrist crease to measure D_0_ and PSV_0_ before ergonovine provocation (Fig. 1). The palce with the most strong brachial dance within the right elbow joint as a puncture point, puncture of the right brachial artery was performed by Seldinger’s Method followed by placing an arterial indwelling needle (220G /1.10 mm × 45 mm, Becton Dickinson Infusion Therapy Systems Inc, Utah, USA) in the brachial artery. Ergonovine (120 µg) was diluted in 5 ml of physiological saline, and then injected into the radial artery within 3 min. One minute later, D_1_ and PSV_1_ after ergonovine provocation were measured by the color Doppler. The inner diameter stenosis degree of the radial artery was calculated according to the formula: [(D_0_–D_1_)/ D_0_] × 100%.

### Safety for the ergonovine provocation test of radial artery

In all patients, blood pressure, heart rate and electrocardiogram were monitored during the ergonovine provocation test of radial artery. Within 5 min after injecting the ergonovine, a small amount of contrast agent was injected into the coronary artery every minute to observe the coronary artery. At the same time, we observed whether the chest pain and/or arrhythmias occurred including sinus bradycardia, second or third degree atrioventricular block, ventricular tachycardia or ventricular fibrillation. If CAS does not relieve consistently during the provocation test of radial artery, intracoronary injection of nitroglycerin should be immediately performed to relieve the CAS.

### Statistical analysis

The measurement data with normal distribution were expressed as mean ± standard deviation, and the measurement data with skewness distribution were expressed as P50 (P25–P75). The enumeration data were expressed as percentage or frequency. The comparisons of data between the two groups were performed using independent sample *t* test, Mann-Whit-ney U test and *χ*^2^ test, respectively. Statistical analysis was performed using SPSS 23.0 software. The predictive value of ergonovine provocation test of radial artery for the diagnosis of CAS was analyzed by receiver operator characteristic (ROC) curve using MedCalc software. Statistical significance was established at *P* < 0.05.

## Results

### Comparisons of general data between the two groups

There were no significant differences in age, sex, diabetes prevalence, LVEF, body mass index (BMI), diastolic pressure, systolic pressure, fasting blood glucose, serum creatinine, blood urea nitrogen, triglyceride, total cholesterol, high density lipoprotein cholesterol (HDL-C), as well as taking aspirin, angiotensin converting enzyme inhibitor (ACEI)/ angiotensin receptor blocker (ARB) and other statins between the two groups (all *P* > 0.05). Low density lipoprotein cholesterol (LDL-C) (*P* = 0.034) and smoking rate (*P* = 0.023) were significantly higher in the CAS group than in the control group (Table 1).

**Table 1:** Comparisons of general data between the two groups.

Items	CAS group (n = 19)	Control group (n = 28)	*P*
Age (year)	57.0 ± 1.2	54.9 ± 1.5	0.347
Sex (male/female)	13/6	18/10	0.769
Smoking (%)	57.9	25.0	0.023
Diabetes (%)	15.8	14.3	0.887
LVEF(%)	63.3 ± 1.1 '	63.0 ± 1.3	0.831
BMI (kg/m^2^)	23.0 ± 1.0	23.5 ± 0.7	0.648
Systolic pressure (mmHg)	130.7 ± 6.1	133.1 ± 3.4	0.715
Diastolic pressure (mmHg)	84.9 ± 3.4	85.5 ± 2.5	0.883
Fasting blood glucose (mM)	5.75 ± 0.19	5.84 ± 0.24	0.804
Serum creatinine (μM)	66.5 ± 3.0	63.7 ± 3.7	0.563
Blood urea nitrogen (mM)	5.04 ± 0.24	5.32 ± 0.29	0.497
Triglyceride (mM)	1.64 ± 0.15	1.75 ± 0.20	0.674
Total cholesterol (mM)	4.60 ± 0.17	4.50 ± 0.13	0.6229
LDL-C(mM)	2.73 ± 0.17	2.35 ± 0.09	0.034
HDL-C(mM)	1.26 ± 0.05	1.27 ± 0.05	0.939
ACEI/ARB (%)	68.4	71.4	0.825
Statins (%)	47.4	50.0	0.859
Aspirin (%)	89.5	85.7	0.705

CAS: coronary artery spasm; LVEF: left ventricular ejection fractio; BMI: body mass index; LDL-C: low density lipoprotein cholesterol; HDL-C: high density lipoprotein cholesterol; ACEI: angiotensin converting enzyme inhibitor; ARB: angiotensin receptor blocker.

### Ergonovine provocation test of radial artery

The degree of radial artery stenosis was significantly higher in the CAS group [41.50% (35.60%, 50.00%)] than in the control group [11.25% (5.15%, 23.00%)] (*P* = 0.000), but there were no siginificant differences in D_0_, PSV_0_ and PSV_1_ between the two groups (*P* > 0.05) (Table 2).

**Table 2:** The results of ergonovine provocation test of radial artery in the two groups.

	D_0_ (mm)	D_1_ (mm)	Radial artery stenosis (%)	PSV_0_ (cm/s)	PSV_1_ (cm/s)
CAS (n = 19)	2.23 ± 0.36	1.47 ± 0.56	41.50%(35.60%,50.00%)	33.4 ± 6.6	30.1 ± 5.9
Control group (n = 28)	2.15 ± 0.36	1.93 ± 0.49	11.25%(5.15%,23.00%)	35.6 ± 7.69	31.5 ± 5.7
*P*	0.430	0.005	0.000	0.311	0.423

The degree of radial artery stenosis is expressed as P50 (P25-P75).

CAS: coronary artery spasm; D_0_: inner diameter of the radial artery before ergonovine provocation; D_1_: inner diameter of the radial artery after ergonovine provocation; PSV_0_: peak systolic velocities of the radial artery before ergonovine provocation and PSV_1_: peak systolic velocities of the radial artery after ergonovine provocation.

### Specificity and sensitivity of the ergonovine provocation test of radial artery for the diagnosis of CAS

**Figure 2:**
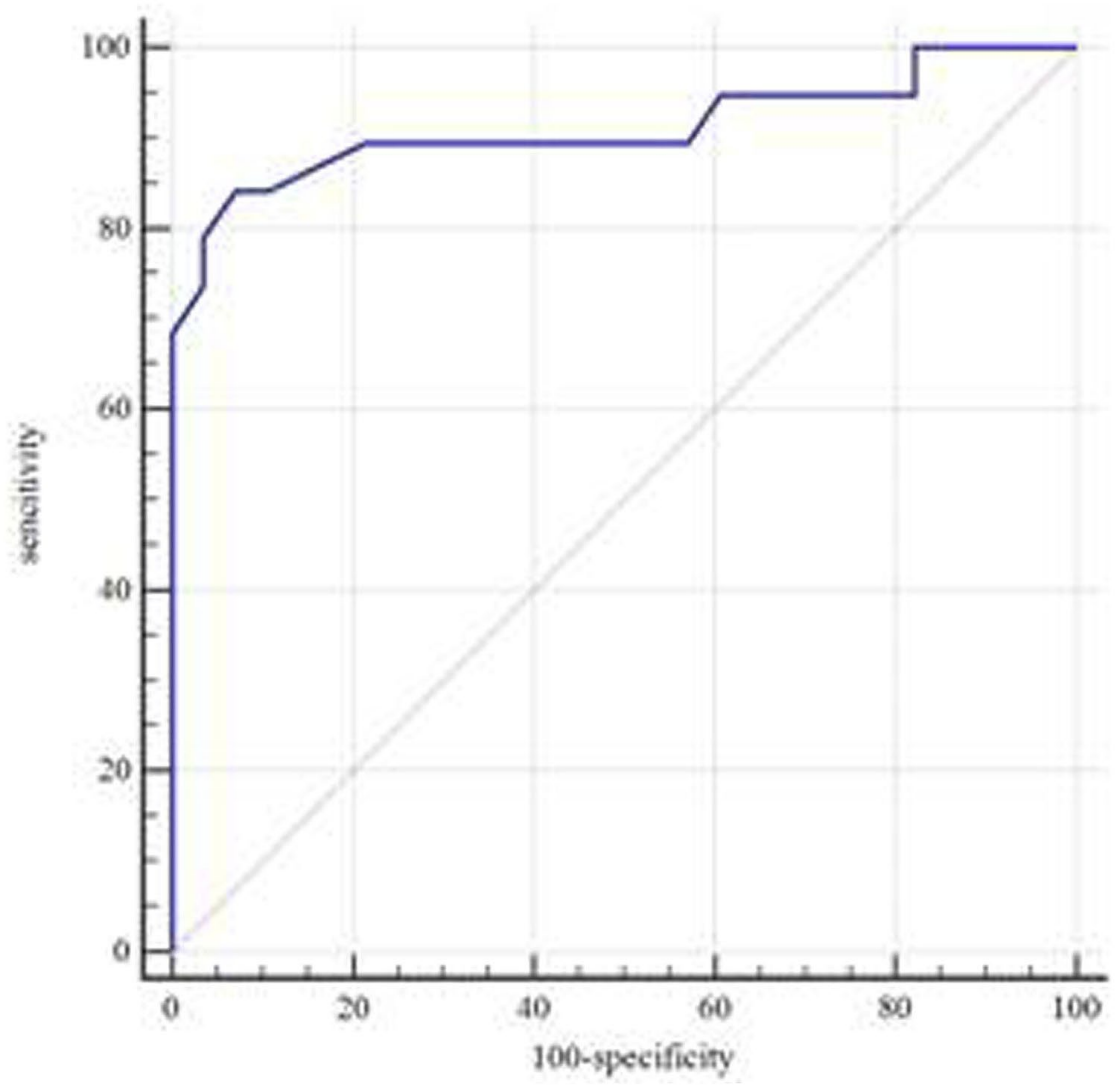
ROC curve of ergonovine provocation test of radial artery for the diagnosis of CAS. *Notes*: ROC: receiver operator characteristic; CAS: coronary artery spasm.

The area under ROC curve of ergonovine provocation test of radial artery for the diagnosis of CAS was 0.912 with 95%CI: 0.792–0.975, *P* = 0.001, cut-off of 31%, specificity of 92.86% and sensitivity of 84.21% (Fig. 2).

### Complications occurring in ergonovine provocation tests of both coronary artery and radial artery

In the intracoronary ergonovine provocation test, one patient had premature ventricular contraction in the control group, as well as one patient had paroxysmal atrial fibrillation and 2 patients had transient hypotension in the CAS group. All these complications disappeared after stopping ergonovine injection, and no fatal or severe complications such as persistent ventricular tachycardia, ventricular fibrillation and myocardial infarction occurred.

The ergonovine (120 µg) provocation test of radial artery did not cause any complications such as coronary artery stenosis, chest pain, electrocardiogram-ischemic change, arrhythmia and hypotension.

## Discussion

The exact mechanism of CAS has not been fully clear. It has been reported that some factors such as smooth muscle hyperresponsiveness, vascular endothelial dysfunction, oxidative stress and inflammatory response are associated with CAS^12–16^. Smoking and hyperlipemia can induce oxidative stress of vascular wall and are the independent risk factors of CAS^17–19^. It has been reported that the smoking rate is higher in the CAS patients than in the non-CAS patients and smoking is a predictor of CAS^20^. He et al.^21^ have described that low density lipoprotein (LDL) is significantly higher in CAS group than in control group. This study found that the smoking rate and LHD-C were significantly higher in the CAS group than in the control group. Our results were similar to previous reports.

The radial artery, α-smooth muscle characteristic artery, has fine vessel diameter and is more sensitive to catecholamine in blood circulation^22^, so placing guide wire, sheathing canal and catheter easily cause vasoconstriction, affecting the experimental results. In this study, the puncture point was located in the brachial artery, an arterial indwelling needle was inserted, and ergonovine was injected into the radial artery by the brachial artery. The puncture point was far away from the radial artery and the diameter of the artery indwelling needle was small, which effectively avoided radial artery spasm induced by mechanical stimulation and ensured the accuracy of the experimental results.

Both radial artery and coronary artery are prone to spasm, and have many same independent predictors^8,23^. In this study, the ergonovine provocation test of radial artery indicated that the degree of radial artery stenosis was significantly higher in the CAS group than in the control group, suggesting that there was a elevation of basic tension in the radial artery of CAS patients. The area under ROC curve of ergonovine provocation test of radial artery for the diagnosis of CAS was 0.912 with cut-off of 31%, specificity of 92.86% and sensitivity of 84.21%, suggesting that the ergonovine provocation test of radial artery is valuable to the diagnosis of CAS due to high specificity and sensitivity. In this study, the degree of radial artery stenosis was significantly higher in the CAS group than in the control group, but PSV_1_ was not significantly different between the two groups. It has been reported that hypervelocity of blood flow is an important index for ultrasound diagnosis of vascular stenosis, but diameter stenosis < 50% usually does not show obvious hemodynamic changes^24^. In this study, the degree of radial artery stenosis was 41.50% (35.60%, 50.00%) in the CAS group, suggesting that the radial artery stenosis caused by 160 µg ergonovine was mild. Therefore, there was no significant different in PSV_1_ between the two groups and PSV has no value in the diagnosis of CAS.

For the intracoronary ergonovine provocation test, initially, 400 µg of ergonovine was injected by peripheral vein, which may cause bilateral coronary artery spasm at the same time and easily lead to serious complications due to intravenous systemic administration^25,26^, so the intravenous administration has been replaced by intracoronary administration. In this study, the ergonovine (120 µg) provocation test of radial artery did not cause CAS, chest pain and electrocardiogram-ischemic change, suggesting that the administration by brachial artery also allowed the ergonovine to enter the bilateral coronary arteries at the same time and 120 µg of ergonovine was more than the dose of ergonovine required by intracoronary ergonovine provocation test, but the final concentration of the 120 µg ergonovine diluted by systemic circulation and pulmonary circulation was not enough to induce CAS, so administration of ergonovine (120 µg) by brachial artery is safe.

CAS is not uncommon clinically. In Japan, 40.9% of angina pectoris was caused by CAS^27^ and 25% of acute coronary syndrome was related to CAS^28^. Intracoronary drug provocation test is the gold standard for the diagnosis of CAS, but it is expensive, requires high technical level and may cause complications, so it is only carried out in a few experienced heart centers. This limits its wide application in clinical practice. Intracoronary drug provocation test is invasive, so patients can not receive this test repeatedly, so this test can not be used for follow-up as well as efficacy evaluation and choice of drugs. We explored the diagnostic value of ergonovine provocation test of radial artery for the diagnosis of CAS by ultrasound for the first time, and results suggested that it may be used as a screening method for CAS, because this method has high sensitivity and specificity; and is simple, safe, non-invasive and non-radiation injured.

The limitation of this study is that the sample size is small. It is necessary to increase the sample size and accumulate more clinical evidence in future studies.
